# Mathematical Model of Blood Circulation with Compression of the Prototype’s Mechanical CPR Waveform

**DOI:** 10.3390/bioengineering9120802

**Published:** 2022-12-14

**Authors:** Xingyuan Xu, Shaoping Wang, Shangyu Wang, Guiling Liu

**Affiliations:** 1 School of Automation Science and Electrical Engineering, Beihang University, Beijing 100191, China; 2 Beihang Ningbo Research Institute, Ningbo 315800, China

**Keywords:** novel CPR prototype, mechanical waveform compressions, cylinder kinetic model, blood circulation model

## Abstract

The waveform of chest compressions directly affects the blood circulation of patients with cardiac arrest. Currently, few pieces of research have focused on the influence of the cardiopulmonary resuscitation (CPR) device’s mechanical waveform on blood circulation. This study investigates the effect of the mechanical waveform from a novel CPR prototype on blood circulation and explores the optimal compression parameters of the mechanical waveform to optimize blood circulation. A novel CPR prototype was designed and built to establish a kinetic model during compressions. The prototype’s mechanical waveforms at various operating conditions were obtained for comparison with manual waveforms and the investigation of the optimal compression parameters. The novel CPR prototype can complete chest compressions quickly and stably. The cardiac output (CO), coronary perfusion pressure (CPP), and cerebral flow (CF) obtained by mechanical waveform compressions (1.22367 ± 0.00942 L/min, 30.95083 ± 0.24039 mmHg, 0.31992 ± 0.00343 L/min, respectively) were significantly better than those obtained by manual waveform compressions (1.10783 ± 0.03601 L/min, 21.39210 ± 1.42771 mmHg, 0.29598 ± 0.01344 L/min, respectively). With the compression of the prototype, the blood circulation can be optimized at the compression depth of 50 mm, approximately 0.6 duty cycle, and approximately 110 press/min, which is of guiding significance for the practical use of CPR devices to rescue patients with cardiac arrest.

## 1. Introduction

It has been over 60 years since CPR was proposed to rescue patients with cardiac arrest [1]. The method of CPR is constantly improved with clinical experiments and scientific analysis, and improving the quality of CPR has become a major concern [2,3,4] in recent years. CPR quality is strongly related to the compression waveform during compressions. On the one hand, it is related to the characteristics of the waveform, i.e., trapezoidal wave, sine wave, etc., [5,6,7]. On the other hand, the frequency and duty cycle of the compression waveform are also relevant [8]. Due to physical changes in the body at different periods after cardiac arrest, there also exist corresponding optimal compression waveforms [9].

A CPR device can be regarded as a mechanical chest compression and non-invasive human circulatory support device [10]. It is referred to as a “circulatory support device” (e.g., LUCAS, Thumper, etc.) in the American Heart Association (AHA) guidelines [11]. However, the outcomes of mechanical CPR are not always uniform. In clinical studies, some research has demonstrated positive outcomes of mechanical CPR [12,13], especially in the process of transportation, which can continuously provide stable chest compressions [14,15,16]. Nevertheless, other research found mechanical CPR has no superiority over manual CPR [17,18,19], and even causes greater harm to patients [20,21,22]. How to make mechanical CPR work consistently is an urgent and worthwhile research problem.

In addition to the CPR devices available on the market, self-developed CPR prototypes are not only cheap [23,24] but also allow for convenient control [25,26,27,28]. These researchers focus more on the usability and stability of the prototypes from a mechanical point of view when developing them, ignoring the effects acting on the human body. However, considering the application scenarios of the prototypes, it is particularly important to analyze the effect of their compressions on the circulation of blood in the human body.

The theoretical outcome of mechanical CPR will differ significantly from manual CPR due to various compression waveforms. Meanwhile, the stable compression of mechanical CPR leads to theoretically stable blood circulation [29]. We are interested in ways to make mechanical CPR stable and work optimally.

Based on the above analysis, the main contributions of this study are as follows:

1. Development of a fast and stable novel CPR prototype, which is convenient to control. The prototype can be installed within 25 s and can operate steadily for more than 20 min;
2. Verification of the superiority of mechanical waveforms over manual waveforms in CO, CPP, and CF, from both mathematical models and experimental results;
3. Exploration of optimal parameters for the prototype’s mechanical waveforms during compressions. We achieved optimal blood circulation by setting the compression depth of this prototype to 50 mm, the duty cycle to 0.6, and the frequency to 110 press/min.

We independently developed a novel CPR prototype, established a mathematical model of the compression process, and validated our ideas through semi-physical experiments, which were instructive for the practical use of CPR devices to rescue patients with cardiac arrest.

## 2. Materials and Methods

### 2.1. Development of a Novel CPR Prototype

According to the basic requirements of the AHA for chest compressions [11], and considering stability and convenience [10], a new CPR prototype was designed in this study.

#### 2.1.1. Structural Design

**Figure 1:**
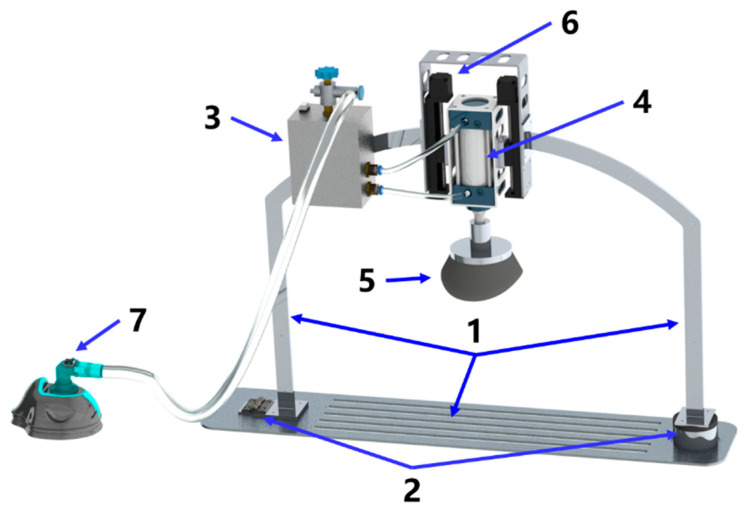
Novel CPR prototype.

The structure of the novel CPR prototype is shown in Figure 1, which mainly consisted of the following parts:

- Lightweight support structure (labeled 1 in Figure 1): the thin-walled support frame and lightweight aluminum alloy base plate with a slot were designed so that the patient can maintain a flat position when placed inside the support structure;
- Quick installation mechanism (labeled 2 in Figure 1): the electromagnetic suction mechanism was designed to achieve rapid error-proof installation for users without training and to earn more CPR time for patients;
- Electrical control (labeled 3 in Figure 1): the miniaturized control module was designed to include a controller, drive circuit, solenoid valve, etc., while providing the balance of inertia during usage;
- Adaptive compression head (labeled 4 and 5 in Figure 1): the compression head was designed to fit the human chest adaptively, and the control box controls the reciprocating motion of the piston in the cylinder to achieve chest compression;
- Accessory mechanisms: a height adjuster (labeled 6 in Figure 1) to adjust the initial height of the adaptive compression module so that the compression head fits the thorax before starting CPR, and an oxygen mask (labeled 7 in Figure 1) to provide respiratory management function in conjunction with chest compression.

#### 2.1.2. System Architecture and Control Method

The compression core consisted of a cylinder, an electro-pneumatic regulator, and a 5-port solenoid valve. The respiratory management core was a 3-port solenoid valve.

For respiratory management, oxygen from a source was adjusted to appropriate pressure through the medical oxygen regulator. The driver circuit was instructed by the controller to adjust the on–off of the 3-port solenoid valve to provide the appropriate pressure of oxygen at the required point in time.

For compression, the controller instructed the driver circuit to adjust the opening of the electro-pneumatic regulator and the direction of the 5-port solenoid valve. The difference in air pressure at the two ends of the cylinder piston led to the telescoping of the piston rod to complete the chest compression.

**Figure 2:**
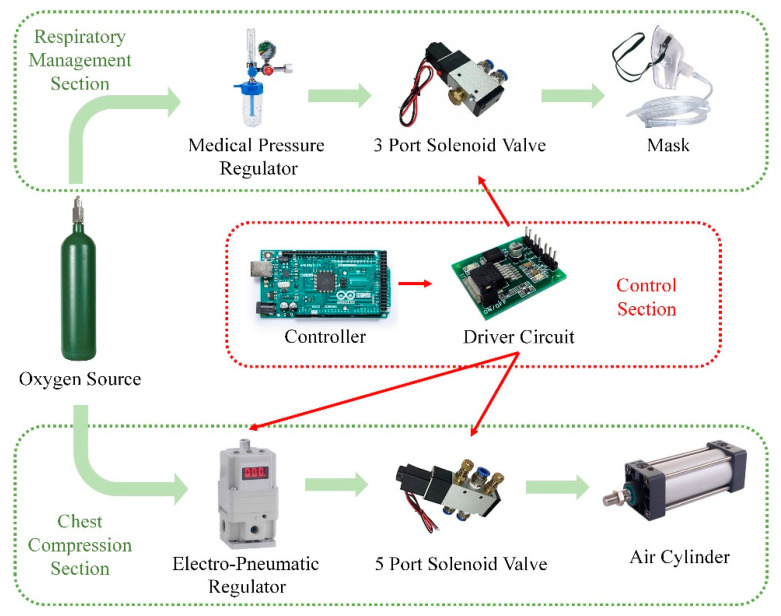
The system architecture of the novel CPR prototype. The green boxes are the respiratory management section and the chest compression section, and the red box is the control section. The green arrow indicates the flow of oxygen, and the red arrow indicates the control signal.

According to the above description, the system architecture of the novel CPR prototype is shown in Figure 2.

To achieve control of the compression depth, frequency, and duty cycle of the chest compression, an appropriate control logic was required to be designed. The compression frequency and duty cycle were controlled directly by the timer in the controller. The compression depth, on the other hand, needed to be closed-loop controlled by the signal from the displacement sensor on the piston. After comparing the error between the extension length of the piston and the set value, a control signal was obtained with the control algorithm. In this closed-loop control, the input signal was the predetermined pressing depth, the output signal was the actual compression depth, and the control algorithm was PID.

### 2.2. Mathematical Model of the Compression Process

A kinetic model of the mechanical CPR compression process was constructed by combining the kinetic model of the CPR prototype with the feedback effect of the human body. By integrating the acceleration of compression in this model, we obtained the velocity of thoracic motion at each time point as the input of the blood circulation model of the human body to estimate the CPR effect.

#### 2.2.1. The Kinetic Model of the CPR Prototype

**Figure 3:**
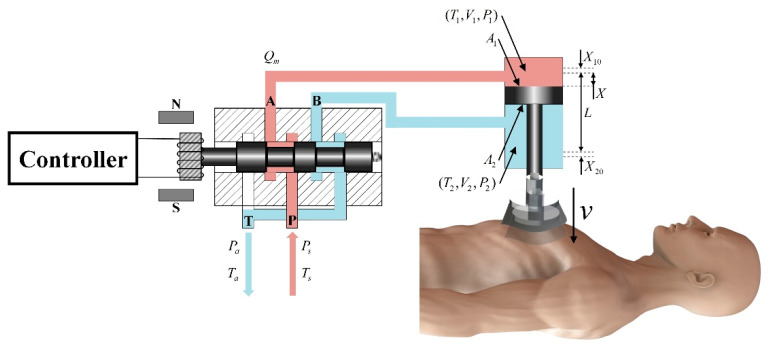
Kinetic model of the power mechanism.

The power mechanism of the CPR prototype was a typical valve-controlled cylinder structure, as shown in Figure 3. The controller emitted a signal to control the displacement of the electro-pneumatic regulator, thereby determining the air pressure. The cylinder was divided into a rodless chamber (working chamber) and a rod chamber (exhaust chamber), and the difference in air pressure between the two chambers led to piston movement to achieve chest compression.

Figure 3 shows the kinetic model of the power mechanism, where *T* is temperature; *P* is pressure; *V* is volume; *X* is piston displacement in the positive direction of extension; *L* is piston stroke; *P*_1_, *V*_1_, and *T*_1_ are rodless chamber parameters; *P*_2_, *V*_2_, and *T*_2_ are rodded chamber parameters; *P*_s_ and *T*_s_ are gas source parameters; *P*_a_ and *T*_a_ are ambiance parameters; *A*_1_ and *A*_2_ are the action area of the piston on the rodless and rodded chamber sides, respectively; and *X*_10_ and *X*_20_ are the equivalent lengths of the initial volumes of the rodless and rodded cavities, respectively.

The following assumptions were made in this model:

(1) The flow at the throttle of the electro-pneumatic regulator was an adiabatic process;
(2) The commutation time of the 5-port solenoid valve was ignored, and it was only considered to have a commutation function;
(3) The inflating and deflating process was rapid in both chambers and the process was adiabatic.

According to the relevant theory of aerodynamics [30], the mass flow *Q_m_* through the throttle of the electro-pneumatic regulator could be obtained as:

(1) $$Q_{m}=c_{0}\eta_{2}A_{0}\frac{p_{u}}{\sqrt{RT}}f_{1}\left(\frac{p_{d}}{p_{u}}\right)$$

where *A*_0_ is the effective open area of the throttle; *p_u_* and *p_d_* are the gas pressures upstream and downstream of the throttle, respectively; *R* is the gas constant; and *c*_0_ is the throttle flow coefficient.

(2) $$f_{1}\left(\frac{p_{d}}{p_{u}}\right)=\{\begin{array}{ll} \frac{\eta_{1}}{\eta_{2}}\sqrt{{\left(\frac{p_{d}}{p_{u}}\right)}^{2/k}-{\left(\frac{p_{d}}{p_{u}}\right)}^{(k+1)/k}} & ,p_{d}/p_{u}>0.528\\ 1 & ,p_{d}/p_{u}\leq 0.528 \end{array}$$

where *k* is the adiabatic index. When the pressure ratio between the downstream and upstream of the throttle was greater than the critical pressure ratio, the flow was subsonic. Otherwise, the flow was sonic and the mass flow reached its maximum. In Equation (2), we define:

(3) $$\begin{array}{cc} \eta_{1}=\sqrt{\frac{2k}{k-1}}, & \eta_{2}=\sqrt{k{\left(\frac{2}{k+1}\right)}^{\left(k+1)/(k-1\right)}} \end{array}$$

The relationship between the effective open area of the throttle *A*_0_ and the electro-pneumatic regulator spool displacement *x_v_* is:

(4) $$A_{0}=x_{v}w$$

where *w* is the area gain. In addition, the effective open area of the throttle *A*_0_ changed with the control voltage *u* as:

(5) $${\dot{x}}_{v}=-\frac{1}{\tau_{v}}x_{v}+\frac{k_{v}}{\tau_{v}}u$$

where $\tau_{v}$ is the response time and $k_{v}$ is the spool displacement gain. Thus, the mass flow at the throttle of Equation (1) could also be written as:

(6) $$Q_{m}=c_{0}\eta_{2}x_{v}w\frac{p_{u}}{\sqrt{RT}}f_{1}\left(\frac{p_{d}}{p_{u}}\right)$$

Therefore, with the control voltage in Equation (5), we calculated the spool displacement and, thus, obtained the mass flow at the throttle in Equation (6). Up until this point, we have established the relationship between the control voltage and the mass flow at the throttle. Following this, the gas passing through the throttle would flow to the two chambers of the cylinder and, thus, drive the piston to move.

The thermodynamic equation for the inflating process of the rodless chamber is:

(7) $$kRT_{s}dM_{s}=V_{1}dP_{1}+kP_{1}dV_{1}$$

where *dM*_s_ is the mass change of the gas in the rodless chamber. The expression of the pressure increments in the rodless chamber was obtained by associating Equation (7), the mass–flow relationship *Q*_1_ = *dM*_s_/*dt*, and the relationship between the rodless chamber volume and piston displacement *V*_1_ = *A*_1_(*X*_10_ + *X*) as:

(8) $$\frac{dP_{1}}{dt}=\frac{kRT_{s}Q_{1}}{A_{1}\left(X_{10}+X\right)}-\frac{kP_{1}}{X_{10}+X}\frac{dX}{dt}$$

Similarly, the thermodynamic equation for the deflating process of the rod chamber is:

(9) $$-kRT_{2}dM_{2}=V_{2}dP_{2}+kP_{2}dV_{2}$$

where *dM*_2_ is the mass change of the gas in the rod chamber. The adiabatic deflating process of finite gas volume is isentropic, so the temperature of the gas in the chamber is:

(10) $$T_{2}=T_{s}{\left(\frac{P_{2}}{P_{s}}\right)}^{\frac{k-1}{k}}$$

The expression of the pressure incremental of the rodded chamber was obtained by associating Equations (9) and (10), the mass–flow relationship *Q*_2_ = *dM*_2_/*dt*, and the relationship between rod chamber volume and piston displacement *V*_2_ = *A*_2_(*L* + *X*_20_ − *X*) as:

(11) $$\frac{dP_{2}}{dt}=\frac{kP_{2}}{L+X_{20}-X}\frac{dX}{dt}-\frac{kRT_{s}Q_{2}}{A_{2}\left(L+X_{20}-X\right)}{\left(\frac{P_{2}}{P_{s}}\right)}^{\frac{k-1}{k}}$$

By integrating the pressure increment of the rodless chamber and the rod chamber, the chambers’ pressure at each moment was obtained. When it satisfied *a*_0_*P*_1_*A*_11_ ≥ *P*_2_*A*_2_ + *F*, the piston started to move. During the motion, the piston satisfies:

(12) $$M_{W}\ddot{X}=a_{0}P_{1}A_{1}-P_{2}A_{2}-F$$

where 0 ≤ *X* ≤ *L*; *M*_W_ is the mass of the piston and other driving parts; *a*_0_ is the effective coefficient of the piston action area on the side of the working chamber; *a*_0_ = 0.8 when *X* = 0; *a*_0_ = 1 when *X* > 0; and *F* in the equation is the resultant force acting on the piston except for the compressed air and can be split as *F* = *F*_1_ ± *F*_2_ ± *F*_3_ ± *P_a_* (*A*_1_ − *A*_2_), where *F*_1_ is the frictional resistance, *F*_2_ is the effective resistance, and *F*_3_ is the component of gravity of the piston and other driving parts in the direction of piston motion. During the compression ($\dot{X}$ > 0) and decompression ($\dot{X}$ < 0) of the chest, the specific expression of *F* is:

(13) $$F=\{\begin{array}{c} \begin{array}{cc} F_{1}+F_{2}-F_{3}+P_{a}\left(A_{1}-A_{2}\right), & \dot{X}>0 \end{array}\\ \begin{array}{cc} -F_{1}+F_{2}-F_{3}+P_{a}\left(A_{1}-A_{2}\right), & \dot{X}<0 \end{array} \end{array}$$

The gas flowing to the two chambers of the cylinder changes the air pressure *P*_1_ and *P*_2_, and with the combined action of force *F* in Equation (13), the acceleration of the piston at each time point can be calculated.

The cylinder and electro-pneumatic regulator parameters used in this section are shown in Table A1 in Appendix A.

#### 2.2.2. Sternal Force Feedback Model

**Figure 4:**
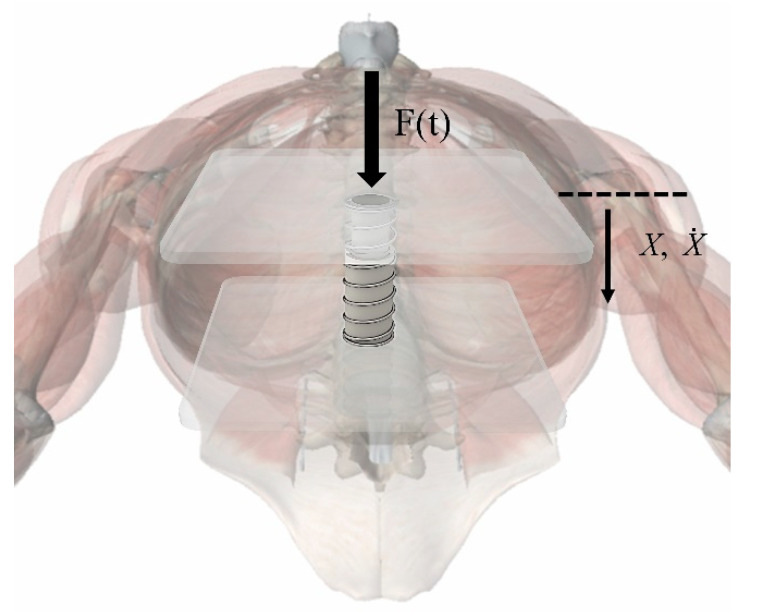
Simplified model of the thoracic cavity.

The feedback of the human sternum and the thoracic internal tissues in response to external forces is highly nonlinear [31,32]. In this study, it was simplified to a primary elastic damping system as shown in Figure 4, and its motion relationship can be described as:

(14) $$F(t)-k_{1}X-m\dot{X}=0$$

where *F*(*t*) is the force applied to the chest, *X* is the chest displacement, $\dot{X}$ is the velocity of the chest, *k*_1_ is the spring elasticity coefficient, and *m* is the damping factor.

The following assumptions were made in this model:

(1) The action point of the external force *F*(*t*) was located directly above the spring and did not change, and its action direction was perpendicular to the chest;
(2) The chest only had a vertical degree of freedom under the action of external force.

After measurement and calculation, the spring elasticity coefficient was *k*_1_ = 7028.9 N/m and the damping factor was *m* = 438.1 Ns/m.

The feedback force of the sternum, in this part, is exactly a part of *F* (*F*_2_) in Equation (12). By calculating this value, the piston acceleration at the current point in time was solved. Since the piston maintains the same motion as the sternum during chest compression, the velocity and displacement of the sternum was obtained by integrating $\ddot{X}$, which, in turn, updated the feedback force of the sternum. With the feedback force constantly updated, we obtained the piston acceleration during the chest compression.

#### 2.2.3. Human Blood Circulation Model

**Figure 5:**
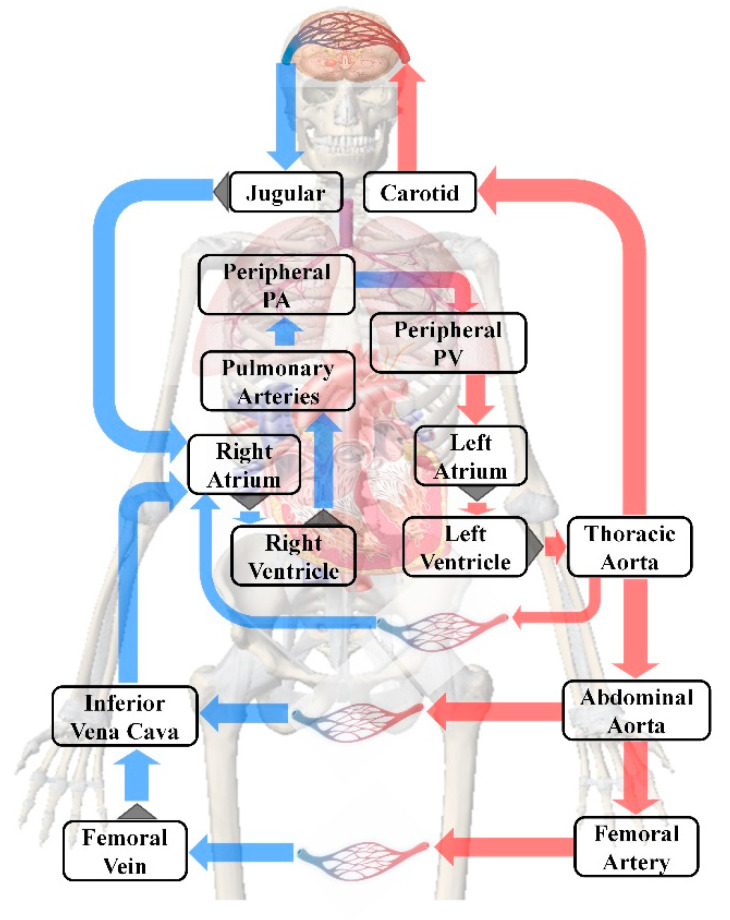
Schematic diagram of the human blood circulation model.

The human blood circulation system is a complex nonlinear system, and to study it quantitatively, we carried out our analysis based on [33]. The human body can be idealized into 14 chambers as shown in Figure 5, in which the blocks represent different chambers and the gray triangles represent valves between chambers.

**Figure 6:**
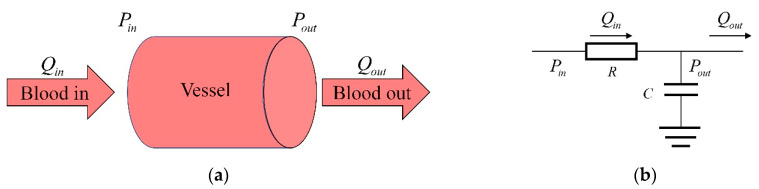
Vessel and its corresponding circuit: (**a**) vessel physiological model, (**b**) vessel corresponding circuit.

In the process of blood flow, the vessel wall expands and contracts with the change of pressure, which is the compliance of the vessel and can be quantified as:

(15) $$C=\frac{\Delta V}{\Delta P}$$

where *C* is the compliance, ΔV is the volume change, and ΔP is the pressure change. During the flow of blood, the flow impedance and compliance given by the blood vessel are similar to the resistance and capacitance characteristics in a circuit, so vessels (Figure 6a) can be abstracted as circuits (Figure 6b).

Where *P_in_* and *P_out_* are the blood pressure at both ends of the vessel, and *Q_in_* and *Q_out_* are the flow at both ends of the vessel. *R* and *C* are the resistance and compliance of the vessel, respectively. The blood flow *Q_in_* can be calculated as:

(16) $$Q_{in}=\left(P_{out}-P_{in}\right)/R$$

The blood pressure can be calculated as:

(17) $$\frac{dP_{out}}{dt}=\left(Q_{in}-Q_{out}\right)/C$$

**Figure 7:**
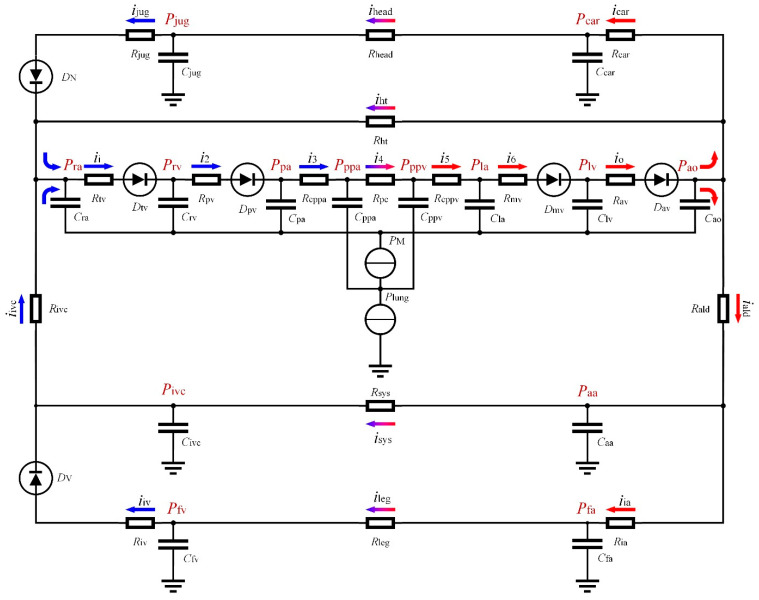
Circuit diagram of the human blood circulation model.

Changing the human blood circulation model in Figure 5 into the lumped model represented by the circuit as shown in Figure 7, we calculated the blood flow following Ohm’s law in the circuit (*i* = △*P/R*).

In Figure 7, all chambers are represented by resistance and capacitance, respectively, and the unidirectional flow of the human valve is characterized by a diode. The arrows in Figure 7 indicate blood flow, where red arrows are arterial blood and blue arrows are venous blood.

The definitions and values of the parameters in the circulation model are shown in Table A2 in Appendix A. The power sources *P*_M_ and *P*_lung_ of the circuit in Figure 7 are derived from chest compressions during CPR, where *P*_M_ is caused by the increasing pressure in the chest and *P*_lung_ is caused by lung compression. The differential of *P*_M_ is calculated as:

(18) $$dP_{M}=\frac{E\left(\dot{X}+{\dot{x}}_{2}\right)}{d_{0}}dt$$

where *E* is Young’s modulus of the chest, $\dot{X}$ is the velocity of the chest, and $\dot{x_{2}}$ is the expansion speed of blood vessels caused by the change of blood volume; *d*_0_ is the thickness of the anterior and posterior tissues of the heart.

The differential of *P*_lung_ is calculated as:

(19) $$dP_{\mathrm{lung}}=\frac{\dot{X}A_{L}-\frac{P_{\mathrm{lung}}-P_{\mathrm{mouth}}}{R_{\mathrm{airway}}}}{C_{\mathrm{lung}}}dt$$

where $\dot{X}$ is the velocity of the chest; *A*_L_ is the cross-sectional area of the compressed lung; *P*_lung_ and *P*_mouth_ are the pressure of lung and mouth, respectively; *R*_airway_ is the impedance of gas flow in the airway; and *C*_lung_ is the compliance of lung.

Both *P*_M_ and *P*_lung_ are related to chest velocity. The velocity of the chest was calculated by integrating over the acceleration of the piston $\ddot{X}$ in Equation (12) since the piston and chest are moving synchronously. With this velocity as the input of the blood circulation model, the blood flow was calculated.

### 2.3. Experimental Design

To verify the stability of the CPR prototype, we designed a total of five tests to operate the device for 20 min under standard compression parameters (compression depth of 50 mm, duty cycle of 0.5, and frequency of 100 press/min) and measure the compression depth and the number of compressions. To verify the rapidity of the CPR prototype, we also designed a total of five tests in which two volunteers placed the CPR simulator in the correct position in the prototype and started chest compressions, calculating the spent time. For comparison, we calculated the time taken by the same two volunteers performing the same task on the Thumper 1007.

Based on the standard compression parameters, we changed one of either compression depth, duty cycle, or frequency, keeping the remaining two parameters as standard (e.g., changing the compression depth to 40 mm, keeping the duty cycle at 0.5 and frequency at 100 press/min). The prototype CPR machine was controlled to work in different situations according to different compression parameters. For each operating situation, we performed continuous compressions and collected 10 sets of compression waveforms for analysis. For experiments varying compression depth, we traversed 20 mm to 50 mm at 5 mm intervals. For experiments varying compression duty cycle, we traversed 0.2 to 0.8 at 0.1 intervals. For experiments varying compression frequency, we traversed 90 press/min to 150 press/min at 10 press/min intervals.

### 2.4. Simulation Design

In order to compare the mechanical waveform of the prototype with the manual waveform under standard compression parameters, we fitted a half-sine waveform with 50 points. The actual manual compression is not a standard sinusoidal waveform but has randomness. Therefore, we added ±1 mm random value to each point (25 points in total) of the first half cycle of each manual waveform. We generated a total of 10 sets of half-sine compression waveforms with random values as manual compression waveforms.

In order to verify the correctness of the experiments, we simulated various mechanical compressions. For the simulation varying the compression depth, we traversed from 20 mm to 50 mm with a 1 mm interval. For the simulation varying the compression duty cycle, we traversed from 0.2 to 0.8 with a 0.01 interval. For the simulation varying the compression frequency, we traversed from 90 press/min to 150 press/min with a 1 press/min interval.

After obtaining the compression waveforms under various conditions, we chose to use the cardiac output (CO) [34], coronary perfusion pressure (CPP) [3,4,35], and cerebral flow (CF) [36,37] as the criteria for evaluating the blood circulation effect. CO calculates the blood flow pumped out of the heart per minute, CPP calculates the pressure gradient between the aorta and the right atrium during the diastolic period, and CF calculates the blood flow of the brain per minute.

## 3. Results

### 3.1. Effects of the CPR Prototype

In the prototype stability validation experiment, it delivered an average of 1999 ± 2.19 compressions per experiment, and each compression reached the required depth. In the rapidity validation experiment, the average time taken by the two volunteers was 24.8 ± 1.47 s in the prototype setup and 25.2 ± 1.47 s on the Thumper 1007, which was not significantly different (*p* > 0.05).

**Figure 8:**
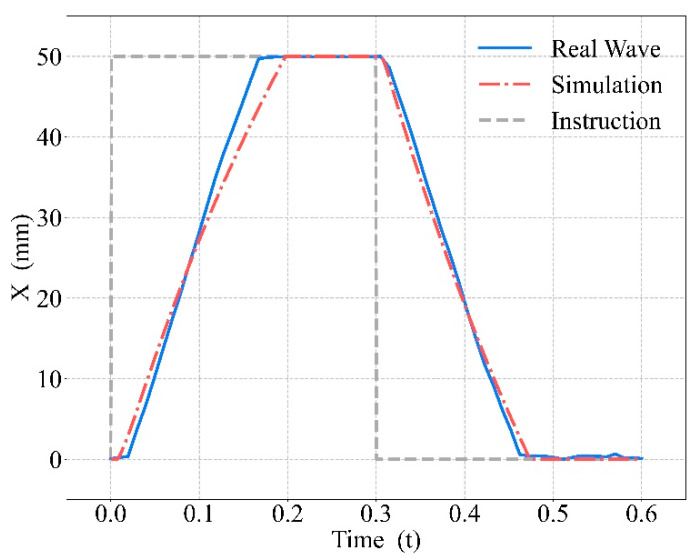
Mechanical waveform, simulated waveform, and the instruction signal.

Driven by the standard compression parameters, the mechanical waveform of the CPR prototype, simulated waveform, and instruction signal are shown in Figure 8. The piston movement characteristics are shown in Table 1. In Figure 8, the blue solid curve is the mechanical waveform, the red dot–dash curve is the simulated waveform, and the gray dashed curve is the instruction signal. For the square wave-like instruction signal, both the simulated and mechanical waveforms resembled trapezoids and had a high degree of overlap.

**Figure 9:**
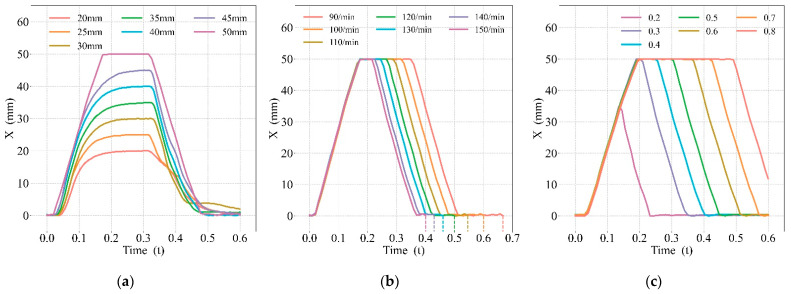
Prototype mechanical waveforms under various instructions: (**a**) various depths, (**b**) various frequencies, (**c**) various duty cycles.

The output curves of the CPR prototype were obtained under the control commands of different compression depths, duty cycles, and frequencies, as shown in Figure 9. Figure 9a shows the experimental results of varying compression depths, Figure 9b shows the experimental results of varying compression frequencies, and Figure 9c shows the experimental results of varying compression duty cycles. The movement characteristics under various instructions are shown in Table 2.

**Figure 10:**
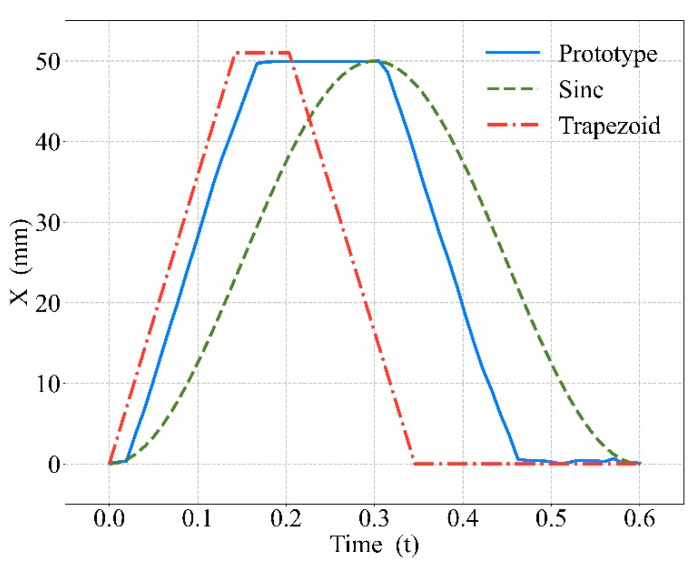
Various compression waveforms.

Current studies use sine or trapezoidal waves as the ideal compression curves, which are significantly different compared with the output waveform of the CPR prototype. Different waveforms are shown in Figure 10, in which the blue solid curve is the output of the prototype used in this study, the green dashed curve is the sine wave [38], and the red dot–dash curve is the trapezoidal wave [9]. Although the trend of the prototype output was similar to the trapezoidal wave, there was a delay in the prototype output and a longer time pressed at maximum depth, which would also lead to better blood circulation.

### 3.2. Comparison between Mechanical and Manual Compression

**Figure 11:**
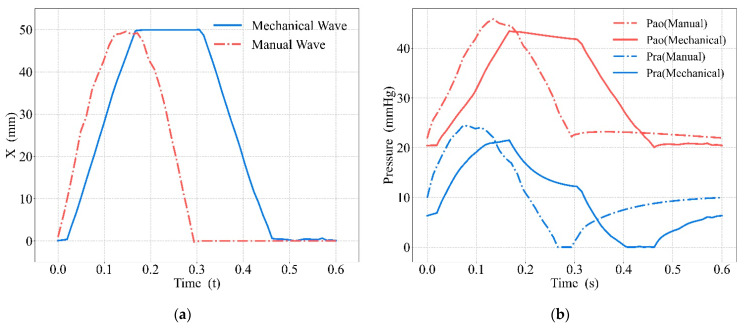
Comparison between mechanical and manual compressions: (**a**) compression waveform, (**b**) blood pressure.

Driven by the standard compression parameters, we compared the compression waveforms and their corresponding changes in aortic and right atrial blood pressure during the compression cycle, as shown in Figure 11. The detailed blood pressure information is shown in Table 3.

The blue solid curve in Figure 11a is the prototype’s mechanical waveform, and the red dot–dash curve is the manual waveform. In Figure 11b, the red curve shows the aortic blood pressure (Pao), the blue curve shows the right atrial blood pressure (Pra), the solid curve describes the blood pressure under mechanical waveform compression, and the dot–dash curve describes the blood pressure under manual compression.

**Figure 12:**
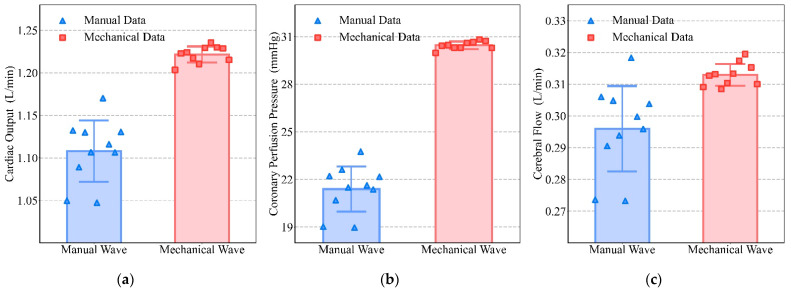
Comparison of blood circulation between mechanical and manual compression: (**a**) CO, (**b**) CPP, (**c**) CF.

Driven by the manual waveform and the mechanical waveform, we obtained the blood circulation, shown respectively in Figure 12 as the CO results (Figure 12a), the CPP results (Figure 12b), and the CF results (Figure 12c). In Figure 12, the blue bars show results under the manual waveform, and the red bars show results under the mechanical waveform. The blue triangular points show each blood circulation outcome when compressed with 10 manual waveforms, and the red square points show each blood circulation outcome when compressed with 10 mechanical waveforms.

It can be seen from the results in Figure 12 that the mechanical waveform was significantly better than the manual waveform in all three evaluation criteria. For CO, the manual waveform was 1.10783 ± 0.03601 L/min, while the mechanical waveform reached 1.22167 ± 0.00942 L/min (*p* < 0.01). For CPP, the manual waveform was 21.39210 ± 1.42771 mmHg, while the mechanical waveform reached 30.45083 ± 0.24039 mmHg (*p* < 0.01). For CF, the manual waveform was 0.29598 ± 0.01344 L/min, while the mechanical waveform reached 0.31292 ± 0.00343 L/min (*p* < 0.01).

### 3.3. Optimal Blood Circulation Results of Mechanical Compressions

**Figure 13:**
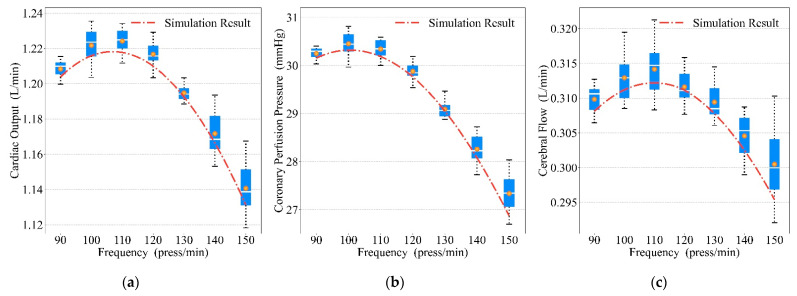
Relationship between frequencies and blood circulation results: (**a**) CO, (**b**) CPP, (**c**) CF.

**Figure 14:**
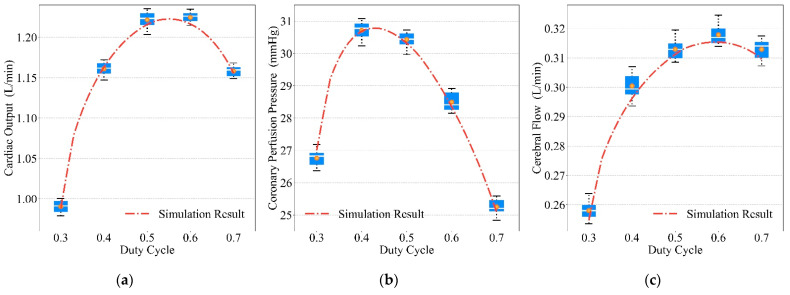
Relationship between duty cycles and blood circulation results: (**a**) CO, (**b**) CPP, (**c**) CF.

**Figure 15:**
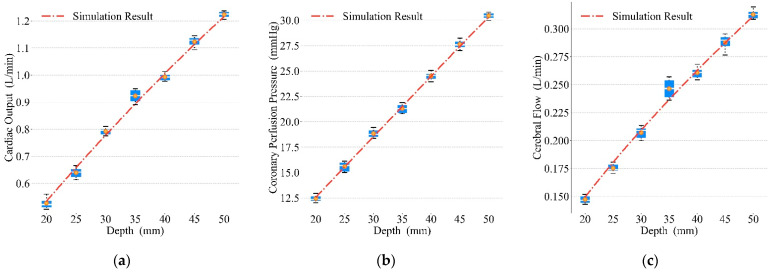
Relationship between depths and blood circulation results: (**a**) CO, (**b**) CPP, (**c**) CF.

We varied the compression frequencies, duty cycles, and depths to obtain the prototype’s mechanical waveforms and the simulated waveforms for chest compression, and the results are shown in Figure 13, Figure 14 and Figure 15. The red dot–dash curves in the figures are the blood circulation of the simulated waveforms. The box describes the blood circulation of the mechanical waveform. The white solid curve in the box is the median value and the orange dot is the mean value. The optimal compression parameters of mechanical waveform and simulated waveform results are shown in Table 4. Each group of data in the table consists of the blood circulation result and its corresponding compression parameter, where the compression parameter is in parentheses (e.g., the data 1.2241 (110) in the first column of the first row indicates that the CO of the mechanical waveform reached a maximum value of 1.2241 L/min at a compression frequency of 110 press/min). As both compression frequency and duty cycle increased, the blood circulation reached optimal value and then gradually became worse. For compression depth, the blood circulation was gradually optimized with the increasing depth.

## 4. Discussion

### 4.1. Effects of the CPR Prototype

From the experimental data, it can be seen that the CPR prototype achieved stable compressions and quick installation. The stability of compression was attributed to the solid structure of the prototype and the continuous operation of the controller and cylinder. The quickness benefited from the quick installation mechanism marked as label 2 in Figure 1.

From the three curves in Figure 8, it can be seen that compared with the instruction signal, there was a delay in both the simulated waveform and the mechanical waveform, which was longer for the compression period, as the thorax provides resistance to the piston during the compression period and power during the decompression period, which leads to a difference in the delay time and the velocity of the piston movement. Since the mechanical waveform overlaps well with the simulated waveform, it can be demonstrated that the mathematical model we established for chest compression was very similar to reality.

For the compression depth results in Figure 9a, the prototype achieved various depths of compressions within the range of the cylinder stroke. Since the stroke of the cylinder used in the experiment was 50 mm, if the compression depth was less than 50 mm, the piston moved more gently due to the control of the PID algorithm. If the compression depth was equal to 50 mm, the throttle of the electro-pneumatic regulator was maximum, so the piston moved at a greater velocity. During the decompression period, the throttle of the electro-pneumatic regulator was maximum at all compression depths, so the piston moved at the maximum velocity at all compression depths.

Since the piston was subjected to resistance related to the velocity of movement, there was a maximum operating speed driven by the air source at a certain pressure. Therefore, after changing the compression duty cycle and frequency, the piston had almost the same speed in the compression and decompression periods, respectively. For various compression frequencies in Figure 9b, the prototype achieved complete chest compressions. For various compression duty cycles in Figure 9c, complete chest compressions were achieved between 0.3 and 0.7.

In Figure 10, the compression curves in other studies [9,38] were ideal compression cases, which were smooth and did not take into account the impact of actual operation. The compression curves with the CPR device were affected by various factors such as the air source pressure and cylinder type, so the mechanical waveforms used in this study were more closely matched to the actual compressions.

### 4.2. Comparison between Mechanical and Manual Compression

It can be seen from Figure 11a that, although the duty cycle was 0.5, there was a great difference in the waveform. During manual compression, the whole period of compression and decompression is considered as the compression period, while for mechanical compression, only the action stroke of the piston is considered as the compression period and the chest is still being compressed during the decompression period. Therefore, although with the same duty cycle, the actual compression time of mechanical waveforms will be longer than for manual waveforms. As can be seen in Figure 11b and Table 3, although the blood pressure is higher with manual compressions, the mechanical compressions cause the aortic pressure to remain at a higher level for a longer time and more blood will be squeezed out, which will lead to better blood circulation. Other experimental results also show that blood circulation will be optimized with a sustained compression time of 0.1–0.2 s [8]. This was also evidenced by the results obtained in Figure 12. Meanwhile, the distribution of data points in Figure 12 can indicate that the effect of mechanical compression was more concentrated and its compression stability was stronger [10]. The effects of manual compressions were more random. Some effects were similar to those of mechanical compressions, while others were inferior. The stability of compressions is important for patients to return to spontaneous circulation [39]. The stability of mechanical compressions has been demonstrated in other research [40] as an inherent advantage of mechanical compressions.

### 4.3. Optimal Blood Circulation Results of Mechanical Compressions

From Figure 13, Figure 14 and Figure 15, similar blood circulation effects can be obtained using the simulated waveforms and the prototype’s mechanical waveforms, which benefit from the accuracy of our mathematical model.

It can be seen from the results in Figure 13 that the increase in compression frequency did not continuously optimize the blood circulation result, and either a too large or too small frequency will worsen the blood circulation, which is similar to the conclusion obtained in [6]. This was similar to our results, but there may be a discrepancy in the precise optimal compression frequency due to differences in the compression waveform.

It can be seen from the results in Figure 14 that the optimal compression duty cycle was achieved around 0.4 based on CPP, but around 0.6 based on CO and CF. Therefore, when the compression duty cycle is greater than 0.5 and reaches about 0.6, a better blood circulation result can be obtained. When using a CPR device to perform compressions, better blood circulation can be achieved when the compression duty cycle is larger [41].

It can be seen from the results in Figure 15 that, the deeper the compression depth is, the better the blood circulation result will be. The relationship between depth and blood circulation results is almost proportional in all three evaluation criteria. The requirement of compression depth shows an increasing trend with the instructions proposed by the AHA guidelines in recent decades, and clinical results have shown an important correlation between compression depth and survival rate [42,43]. However, blindly increasing the depth of compressions will cause damage to the human skeleton [44], and the same compression depth is not suitable for patients [45]. Therefore, it is necessary to increase the compression depth within a reasonable range.

The optimal compression parameters and their corresponding acquisition methods in other studies are shown in Table 5 below.

Studies [38,46,47,48] all used sine waves for compressions, while [9] used the trapezoidal wave, which is similar to the mechanical CPR waveform in this study. For the compression depth in all studies, it is reflected that increasing within a reasonable range will optimize blood circulation. For compression frequencies and compression duty cycles, there exist differences between the results of different studies. For the compression frequencies, most results show that around 110 press/min will achieve better blood circulation. For the compression duty cycles, the studies point out that compressions with sine waves optimize blood circulation at less than 0.5, whereas with trapezoidal waves, which are similar to the prototype mechanical waveform, the optimal duty cycle is greater than 0.5. Since the faster compression velocity will lead to better blood circulation [2,49], the sine wave will produce a faster compression velocity when the duty cycle is smaller. The trapezoidal waveform is similar to the mechanical waveform where compressions of maximum depth are sustained for a period, which will optimize blood circulation [8]. Therefore, a larger duty cycle is required in the mechanical waveform.

There is a discrepancy between the optimal compression parameters with the mechanical waveform of the CPR prototype and those with manual compressions, which may lead to the inability to achieve optimal CPR results when designing the CPR device. The blood circulation in this study relies on the output of the blood circulation model, and further demonstration of the results will require additional clinical experiments.

## 5. Conclusions

In this study, we designed and manufactured a fast and stable novel CPR prototype, and, based on it, explored the blood circulation result of chest compression. Experiments showed that when offering chest compressions, the prototype’s mechanical waveform obtained a better blood circulation result than the manual waveform. We also investigated the optimal parameters for compressions with the CPR prototype, which is of guiding significance to rescue patients in cardiac arrest with a CPR device.

## Figures and Tables

**Table 1:** Piston movement characteristics (real wave vs. simulation).

	Start to Compress (s)	Fully Compressed (s)	Start to Decompress (s)	Fully Decompressed (s)
Real Wave	0.028	0.165	0.310	0.465
Simulation	0.007	0.198	0.304	0.474

**Table 2:** Piston movement characteristics under various instructions.

Depths	Start to Compress (s)	Fully Compressed (s)	Start to Decompress (s)	Fully Decompressed (s)
20 mm	0.043	0.236	0.317	0.554
25 mm	0.039	0.240	0.319	0.563
30 mm	0.039	0.227	0.330	0.431
35 mm	0.039	0.253	0.328	0.471
40 mm	0.034	0.249	0.328	0.492
45 mm	0.030	0.249	0.322	0.523
50 mm	0.028	0.165	0.310	0.465
**Frequencies**	**Start to Compress (s)**	**Fully Compressed (s)**	**Start to Decompress (s)**	**Fully Decompressed (s)**
90 press/min	0.031	0.171	0.354	0.505
100 press/min	0.028	0.165	0.310	0.465
110 press/min	0.028	0.175	0.293	0.444
120 press/min	0.031	0.168	0.270	0.420
130 press/min	0.030	0.172	0.250	0.399
140 press/min	0.028	0.165	0.229	0.376
150 press/min	0.031	0.171	0.220	0.363
**Duty Cycles**	**Start to Compress (s)**	**Fully Compressed (s)**	**Start to Decompress (s)**	**Fully Decompressed (s)**
0.2	0.032	0.133 (34.4 mm)	0.133	0.223
0.3	0.032	0.177	0.207	0.344
0.4	0.028	0.187	0.252	0.397
0.5	0.028	0.165	0.310	0.465
0.6	0.031	0.181	0.362	0.505
0.7	0.031	0.181	0.429	0.562
0.8	0.032	0.185	0.494	-

**Table 3:** Detailed blood pressure information with mechanical and manual waveforms.

	Aortic		Right Atrial	
	Maximum Value	Reached Time	Maximum Value	Reached Time
Manual	45.88 mmHg	0.135 s	24.44 mmHg	0.074 s
Mechanical	43.47 mmHg	0.167 s	21.50 mmHg	0.167 s

**Table 4:** Optimal parameters for compression.

	Cardiac Output (L/min)		Coronary Perfusion Pressure (mmHg)		Cerebral Flow (L/min)	
	Real Wave	Simulation	Real Wave	Simulation	Real Wave	Simulation
Frequency (press/min)	1.2241 (110)	1.2181 (107)	30.431 (100)	30.318 (100)	0.314 (110)	0.312 (110)
Duty Cycle	1.2237 (0.6)	1.2227 (0.55)	30.715 (0.4)	30.787 (0.43)	0.318 (0.6)	0.315 (0.59)
Depth (mm)	1.2218 (50)	1.2158 (50)	30.431 (50)	30.318 (50)	0.313 (50)	0.311 (50)

**Table 5:** Comparison of optimal pressing parameters.

Study	Depth (mm)	Frequency (Press/min)	Duty Cycle	Methods
Daudre-Vignier [46]	50	137	0.28	Sine waves in lumped model
John [38]	57	110	-	Sine waves in lumped model
Suval [47]	47	107	-	Sine waves in clinical experiments
Nas [48]	47	107	-	Sine waves in clinical experiments
Lampe [9]	51	125	0.73	Trapezoidal waves in clinical experiments
**Ours**	**50**	**110**	**0.6**	**Mechanical waves in lumped model**

## Data Availability

This study does not report any data.

## Appendix A

**Table A1:** Cylinder and electro-pneumatic regulator parameters.

Parameters	Value
*X*_10_	2.445 × 10^−2^ m
*X*_20_	2.911 × 10^−2^ m
*A*_1_	1.963 × 10^−3^ m^2^
*A*_2_	1.649 × 10^−3^ m^2^
*w*	1 m
$\tau_{v}$	0.004 s
$k_{v}$	1 m/V
*R*	8.314 J/(mol·K)
*k*	1.4
*P_a_*	101,325 Pa
*P_s_*	400,000 Pa
*T*	293 K
*L*	50 mm
*F*_1_	15 N
*F*_3_	18.2 N

**Table A2:** Definitions and values of parameters.

Name	Abbreviation	R (mmHg/L/s)	C (L/mmHg)
Right atrium	ra		0.0095
Right ventricle	rv		0.016
Pulmonary arteries	pa		0.0042
Peripheral pulmonary arteries	ppa		0.0042
Peripheral pulmonary veins	ppv		0.00128
Left atrium	la		0.0128
Left ventricle	lv		0.008
Thoracic aorta	ao		0.0008
Carotid	car	60	0.0002
Jugular	jug	30	0.012
Abdominal aorta	aa		0.0004
Inferior vena cava	ivc	25	0.0234
Femoral artery	fa		0.0002
Femoral vein	fv		0.0047
Head	head	5520	
Pulmonary capillary bed	pc	105	
Tricuspid valve	tv	5	
Mitral valve	mv	5	
Pulmonic valve	pv	10	
Aortic valve	av	10	
Between central and peripheral pulmonary arteries	cppa	10	
Between central and peripheral pulmonary veins	cppv	5	
Coronary vessels	ht	15780	
Aorta	ald	25	
Leg vasculature	leg	8520	
Both iliac arteries	ia	360	
Both iliac veins	iv	180	
Residual systemic vasculature	sys	1800	
Airways to ventilation	airway	1.2	
Lung	lung		0.23
